# Platelet-rich plasma to treat experimentally-induced skin wounds in animals: A systematic review and meta-analysis

**DOI:** 10.1371/journal.pone.0191093

**Published:** 2018-01-11

**Authors:** Adolfo Maria Tambella, Anna Rita Attili, Gilles Dupré, Andrea Cantalamessa, Stefano Martin, Vincenzo Cuteri, Sabrina Marcazzan, Massimo Del Fabbro

**Affiliations:** 1 School of Biosciences and Veterinary Medicine, University of Camerino, Matelica, MC, Italy; 2 Clinic for Small Animal Surgery, Department for Clinical Sciences, Veterinary Medicine University Vienna, Vienna, Austria; 3 Department of Biomedical Surgical and Dental Sciences, University of Milan, Milan, Italy; 4 IRCCS Galeazzi Orthopaedic Institute, Milan, Italy; Massachusetts General Hospital, UNITED STATES

## Abstract

The objective of the study was to review current literature to determine whether the topical application of platelet-rich plasma (PRP) promotes healing in experimentally-induced full-thickness skin wounds in animals. The hypothesis was that the adjunct of PRP has a positive effect on wound healing. An electronic search was carried out on the following databases: Web of Science, Cochrane Library, PubMed, Research Gate, Cochrane Wounds Group, Veterinary Information Network. No publication date nor language restrictions were applied. Randomised and not randomised controlled clinical trials comparing PRP with placebo or with other treatments were included. The reduction of open wound area in PRP-treated (test) wounds compared to control wounds was the primary outcome. Secondary outcomes were healing time and number of healed cases in test group compared to control. The following effect sizes were calculated: the Hedges’ g for continuous variables; the odds ratio for binary data. Eighteen controlled clinical trials were included in the qualitative and quantitative synthesis, with a total of 661 wounds. All studies were published in the period 2007–2016. Eight studies were carried out on rodent/lagomorph mammals and 10 on non-rodent/lagomorph mammals. In all included studies, control wounds underwent placebo or were left untreated. The PRP group showed a better healing performance than the control group in each outcome. The effect size was statistically significant considering the primary outcome and the overall aggregation of the three outcomes. The effect size, although in favour of the treatment with PRP, was not significant considering the healing time and the number of healings. The overall heterogeneity was mild or moderate. Five studies reported a high risk of selection bias. The publication bias was always mild or absent. The results support the hypothesis of the positive effects of the PRP when compared to control groups in the treatment of experimentally-induced full-thickness skin wounds in animals. PRP can therefore be considered an effective adjunctive therapy in stimulating second intention healing of acute wounds in healthy animals.

## Introduction

The wound healing process is usually divided into three main phases: the inflammatory phase, the proliferative phase, and tissue remodeling. These events are regulated by a complex interaction of molecular signals involving mediators, primarily cytokines and growth factors (GFs). The GFs stimulate and modulate the major cellular activities involved in the healing process. In chronic wounds, the normal progression is disrupted and slowed down, so that healing difficulties arise [1–5].

Platelets play a fundamental role in the healing process of skin wounds. The platelet-derived GFs are particularly important during the proliferation phase (fibroplasia, reepithelialisation and neovascularisation), as they are involved in the recruitment of mesenchymal cells, and in the synthesis of the extracellular matrix [4–8].

Following spontaneous or induced activation, platelets release the GFs contained in the alpha granules, including: Platelet-Derived Growth Factor (PDGFαα/ββ/αβ), Transforming Growth Factor β1/2 (TGF-β1/2), Vascular Endothelial Growth Factor (VEGF), Basic Fibroblast Growth Factor (bFGF), Platelet-Derived Epidermal Growth Factor (PDEGF), Insulin-like Growth Factor-I/II (IGF-I/II) [9,10].

Platelet-Rich Plasma (PRP) is a platelet concentrate that is applied locally at the injury site, upon activation. In recent years, different preparation protocols and activating agents have been proposed. All the following substances are considered activating agents: bovine thrombin/thromboplastin, agonist peptide of the thrombin receptor, ITA gelling agent (NATREX Technologies, Inc., Greenville, NC), batroxobin (clotting enzyme isolated from the venom of the snake *Bothrops atrox*, belonging to the Viperidae family), calcium chloride (CaCl_2_), ascorbic acid and autologous thrombin [8,11–16]. Using different methods of preparation, two types of PRP can be obtained: PRP with the presence of leukocytes (L-PRP) and pure PRP (P-PRP), without leukocytes [17–19].

In the recent years the positive effect of PRP for healing enhancement has been reported in many applications of human medicine: skin ulcers (bedsores and diabetic ulcers), plastic-reconstructive and cosmetic surgery [1,8,10,20,21,22,23]; oral-maxillofacial surgery [10,22]; cartilage and tendon repair [10,22]; orthopaedic surgery and bone reconstruction (e.g. delayed union, nonunion, ischemic osteonecrosis, osteolysis, tendon-muscular diseases) [10,22,24,25]; and ophthalmology (corneal ulcers) [10,22]. Despite the growing interest, the scientific literature is still limited in veterinary medicine, where a paucity of randomised clinical trials can be observed [12,26–35].

Before designing clinical studies on large human and animal populations with spontaneous disease, there is the need to assess the evidence of the literature regarding the application of PRP in experimentally-induced wounds in animals.

The objective of the study was to review current literature in order to determine whether topical application of PRP promotes the healing process in experimentally-induced full-thickness skin wounds in animals. The hypothesis was that the adjunct of PRP, compared with placebo or with other treatments, has a positive effect on wound healing.

## Methods

In this systematic review, the principles of the PRISMA guidelines (Preferred Reported Items for Systematic Review and Meta-analyses) [36,37] and the Cochrane Handbook for Systematic Reviews of Interventions [38] were followed. A step-by-step systematic review protocol was deposited in protocols.io (dx.doi.org/10.17504/protocols.io.k5rcy56).

### Criteria for considering studies for this review (*Study eligibility criteria*)

To define the criteria for inclusion of each primary study, a structured approach type PICOS (Population, Intervention, Comparison, Outcomes, Study design) was used. Below, the main features are described.

Randomised and non-randomised controlled clinical trials (CCTs) that compared PRP with other treatments or placebo were considered.

The population under study consisted of animals of all species, breed and age, on which full-thickness skin wounds were experimentally induced, and left to heal by secondary intention.

Studies that compared PRP (produced by any method) with placebo or with other topical therapies such as standard care or biomaterials were eligible for this systematic review.

The **primary outcome** was represented by the reduction in size of open wound area in the PRP treated wounds compared to the size reduction in control wounds (reported in primary studies as: the percentage of reduction of wound area compared with baseline; the percentage of open wound area, or not healed, or not re-epithelialized, compared to the admission; the absolute wound area expressed in cm^2^).

The **secondary oucomes** consisted of:

- healing time (time needed to obtain the complete healing of the wound in PRP treated wounds compared to controls);
- number of healings (proportion of wounds showing complete healing in PRP treated wounds compared to controls).

Any reference to the assessment of wound complications, wound pain, quality of life and adverse events related to the intervention was also sought.

### Criteria for considering publications for this review (*Report eligibility criteria*)

No restriction was placed regarding language and publication date. Only studies published on indexed, peer-reviewed Journals were considered.

### Search methods for identification of studies

The literature search was conducted with broad search criteria, so as to limit the number of false negatives (relevant studies but not found during the search phase), while increasing the number of false positives (studies found during the search phase that do not truly meet the inclusion criteria) [39].

The electronic search was undertaken on the following databases: Web of Science, Cochrane Library, PubMed, Research Gate, Cochrane Wounds Group, Veterinary Information Network (VIN).

In the aforementioned databases, the search was done using the following keywords, combined using the Boolean operators AND, OR:

- platelet / platelet-rich / platelet-rich plasma / platelet gel;
- wound / skin / ulcer;
- animal / dog (canine) / horse (equine) / pig (swine) / goat (caprine) / sheep (ovine) / cow (cattle, bovine) / cat (feline) / rabbit (cunicola) / mouse (mice, murine) / rat.

When it was necessary to obtain additional information, especially in cases of incomplete data, the authors of the clinical studies were contacted directly.

### Data collection and analysis

#### Selection of studies

The screening was performed by two independent reviewers (AMT, MDF). All identified studies were assessed by the inclusion/exclusion criteria then subjected to the screening phase. First of all, duplicates emerging from one or more search strategies and databases have been excluded.

The *records screened* were selected using a two-step approach, first by analyzing the title and abstract (with identification of the # of *records excluded*), then by analyzing the full-text (with identification of the # of *full-text articles assessed for eligibility*). The reason for exclusion was specified for each of the excluded references (# of *full-text articles excluded*, *with reasons*).

Finally, the identified studies were classified as included in the systematic review (# of *studies included in the qualitative synthesis*) and in the meta-analysis (# of *studies included in the quantitative synthesis—meta-analysis*) thus completing the PRISMA flow diagram.

#### Data extraction and management

The following data from each included primary study were extracted and recorded in a data extraction form:

- study characteristics (name, design, country, funding source);
- publication characteristics (year, language, type);
- participants’ characteristics (number, species);
- characteristics of induced lesions (size and number of wounds, induction mode);
- intervention characteristics (PRP production technique, platelet concentration);
- treatment protocol (division into groups and groups description, randomisation, number of PRP applications, frequency of applications, bandage);
- assessments carried out in primary studies (outcome measures, the presence of multiple time points or waves);
- main results of primary studies.

#### Assessment of risk of bias in included studies

The risk of bias assessment was based on the guidance in the Cochrane Handbook of Systematic Reviews of Intervention [38].

The adequacy of the method used to generate the allocation sequence (random sequence generation, selection bias), the method of allocation concealment (allocation concealment, selection bias), the level of blinding (blinding of outcome assessment, detection bias), the presence of incomplete outcome data (attrition bias), and the defect in the reproduction of results (selective reporting, reporting bias) were examined.

#### Measures of treatment effect

For outcomes represented by continuous variables (reduction in size of the wound area, healing time), to statistically measure the effect size (ES), the Hedges’ g was used. It was calculated starting from the Cohen’s d, using the correction coefficient J.

As data entry format arising from primary studies, mean values, standard deviations, and the sample size of both groups were preferentially used (gold data entry format). If the preferential data entry format was not represented in primary studies, a hierarchical scale for data entry format was established, as described below:

1. mean values, standard deviations, and the sample size;
2. mean values, t-value (result of t-test), sample size;
3. mean values, statistical significance (p-value), sample size;
4. t-value, sample size;
5. p-value, sample size.

For binary outcome data (number of healings), to statistically measure the ES, the odds ratio (OR) was used. To calculate the OR the number of subjects healed (event) and the total sample size of each group for each study was used as gold data entry format.

The unit of analysis was the single wound.

The authors of primary studies were contacted in order to obtain additional information where data were missing or unclear.

#### Management of complex meta-analytical databases

In case of detection in primary studies of complex meta-analytical databases, such as independent subgroups, multiple outcomes, multiple comparisons, multiple time points (waves), the complexity of data was maintained in the analysis wherever possible in order to provide the most accurate possible synthesis. Otherwise, the possibility of performing a pre-analysis for each complex database was considered. The pre-analysis permitted the choice between two options: carry out the analysis separately by calculating an ES for each complex database, or aggregate the various meta-data to obtain a unique effect.

#### Assessment of heterogeneity

The presence of heterogeneity was assessed with the Q homogeneity test, in order to assess whether the meta-analysis was characterised by significant heterogeneity. The impact of heterogeneity was statistically quantified using the I^2^.

The I^2^ value was interpreted on the basis of the cut-off proposed by Higgins et al., according to which values equal to 25%, 50% and 75% respectively indicating low, moderate and high levels of heterogeneity [40,41].

#### Assessment of reporting biases

The publication bias assessment was performed using the funnel plot. The symmetry of the funnel plot was statistically tested with Egger’s linear regression method. To compare the observed ES and the estimated ES in the absence of publication bias, the Trim and Fill method was used (the absence of difference between the two types of effect size indicated an absence of risk of publication bias, a minimum difference minimal risk, and so on).

#### Data synthesis

Any statistical analyses of metadata were performed with software ProMeta version 2 (Internovi, Cesena, Italy).

#### Analysis of the moderators and evaluation of heterogeneity

To assess the possible heterogeneity of the studies, in order to explain which factors might affect it, potential moderators of the results obtained have been considered and analyzed, in particular: country (recodified to: Asia, Europe, North America, South America); animal species (recodified to: rodents/lagomorphs and non rodents/lagomorphs.); initial wound size (≥ and < 1 cm^2^); funding source (for profit funding, for non-profit funding and no-funding statement); n. of spinning cycles for PRP production (single or double spin); activation (application and non-application of activation procedures); platelet concentration in PRP (≥ and < 10^6^ plt/microL); n. of treatments (single treatment and multiple treatment).

#### Sensitivity analysis

For each meta-analysis project, a sensitivity analysis was carried out by performing the meta-analysis, by excluding one study at a time.

## Results

### Description of studies

**Fig 1:**
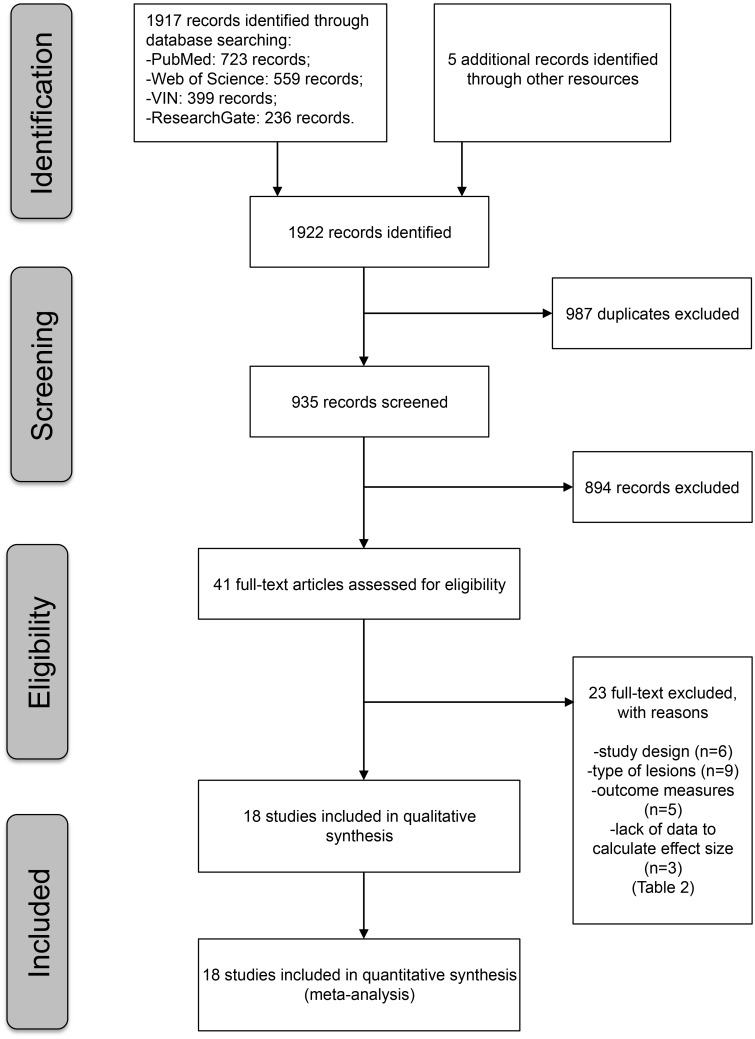
Selection of primary studies: PRISMA flow diagram.

The research performed in the bibliographic databases and references of primary studies identified 1922 documents. After removing duplicates, the first screening based on title and abstract provided 41 eligible studies. The full-text assessment of such studies allowed inclusion of 18 primary studies in both qualitative and quantitative synthesis (meta-analysis) [30,42–58]. (Fig 1)

A total of 661 experimental wounds were considered in this systematic review, with an average of 36.72±37.69 wounds/study (range 8-180/study).

In all included studies, wounds were created by full-thickness skin excision; control wounds underwent placebo (saline solution or milli-Q-water) or were left untreated; treated wounds received L-PRP application. Treated and control wounds were dressed with the same bandage technique.

The main characteristics of primary studies are reported in Table 1.

**Table 1:** Characteristics of the included studies in alphabetical order.

Primary Studies	GD	FS	AS	NW	WS	WD	MW	MP	MA	PC	NT
[42] Abegão 2015	SA	NFS	RL	12	50.2	FT	Punch	DS	-	NR	2
	Brazil		(rabbit)								
[43] Al-Bayati 2013	AS	NS	NRL	40	900.0	FT	Blade	DS	CaCl_2_	<	1
	Iraq		(goat)								
[44] Barrionuevo 2015	SA	NFS	RL	12	50.2	FT	Punch	DS	CG	NR	1
	Brazil		(rabbit)								
[45] Bauer 2009	SA	NS	RL	8	19.6	FT 2mm	Punch	SS	CaCl_2_	<	1
	Brazil		(rat)								
[46] Blanton 2009	NA	P	NRL	48	150.0	FT 7.9mm	Blade	SS	T/TP CaCl_2_	<	1
	USA		(pig)			ep,de,sc					
[47] De Souza 2015	SA	NP	NRL	28	625.0	FT	Blade	DS	-	<	1
	Brazil		(horse)			ep,de,sc					
[48] Dionyssiou 2013	EU	P	RL	40	400.0	FT	Blade	SS	-	≥	1
	Greece		(rabbit)								
[49] Hadad 2010	NA	P	NRL	36	150.0	FT 4mm	-	SS	T/TP CaCl_2_	<	1
	USA		(pig)								
[50] Jee 2016	AS	NS	NRL	24	28.3	FT	Punch	SS	-	≥	2
	Korea		(dog)			ep,de					
[51] Karayannop. 2015	EU	P	NRL	36	400.0	FT	-	SS	-	≥	1
	Greece		(dog)								
[52] Lee 2008	NA	NP	RL	30	625.0	FT	Blade	SS	T/TP CaCl_2_	NR	1
	USA		(rabbit)			ep,de,sc					
[53] Monteiro 2009	EU	NP	NRL	24	625.0	FT	Blade	SS	T/TP CaCl_2_	NR	2
	France		(horse)			ep,de,sc					
[54] Nisbet 2009	AS	NS	RL	30	225.0	FT	Blade	DS	T/TP CaCl_2_	≥	2
	Turkey		(rabbit)			ep,de,sc					
[55] Notodihardjo 2014	AS	NP	RL	180	28.3	FT	Punch	DS	CaCl_2_	≥	1
	Japan		(mouse)			ep,de,sc					
[30] Sardari 2011	AS	NP	NRL	30	400.0	FT	-	DS	T/TP CaCl_2_	<	2
	Iran		(dog)								
[56] Vermeulen 2009	EU	NS	NRL	21	400.0	FT 1cm	Blade	DS	T	NR	1
	Belgium		(pig)			ep,de,sc					
[57] Yan 2007	NA	P	NRL	48	226.9	FT 5mm	Punch	DS	T/TP CaCl_2_	NR	1
	USA		(pig)								
[58] Yang 2011	Asia	NP	RL	14	400.0	FT	Scissor	DS	-	NR	1
	Korea		(mouse)			ep,de,sc					

GD: Geographical distribution; SA: SouthAmerica; NA: NorthAmerica; EU: Europe; AS: Asia; FS: Funding source; P: for profit funding; NP: for non-profit funding; NFS: no-funding statement; NS: no statement regarding the funding source; AS: Animal species; RL: rodent/lagomorph mammals; NRL: non-rodent/lagomorph mammals; NW: Number of wounds; WS: Initial wound size (mm^2^); WD: Wound depth; FT: full-thickness; ep: epidermidis; de: dermis; sc: subcutaneous tissue; MW: Method of wounding; MP: Method of PRP production; SS: single spin; DS: double spin; MA: Method of PRP Activation; CG: Calcium Gluconate; T: Thrombin; TP: Thromboplastin; PC: Platelet concentration; <: platelet concentration < 10^6^ plt/microL; ≥: platelet concentration ≥ 10^6^ plt/microL; NR: not reported. NT: Number of treatments

After full-text evaluation, 23 studies were excluded. These are listed in Table 2, along with the reason for their exclusion [12,26,27,32,33,35,59–75].

**Table 2:** Characteristics of excluded studies in alphabetical order.

Studies	Reasons for exclusion
[12] Carter et al, 2003	Histological study
[59] Chung et al, 2015	Case report
[26] DeRossi et al, 2009	Histological study; sutured wounds, primary intention healing
[60] Demidova-Rice et al, 2012	Histological and biomolecular study
[61] Ferdousy et al, 2013	Histological study; sutured wounds, primary intention healing
[62] Henderson et al, 2003	Experimental lesions consist of burns and not by full-thickness wounds
[63] Hermeto et al, 2012	The study considered skin grafts; lack of data to calculate the ESe
[32] Iacopetti et al, 2012	Case report
[27] Kim et al, 2009	Case report
[64] Koempel et al, 1998	Experimental lesions on tracheal mucosa
[65] Lian et al, 2014	Lack of data needed to calculate the effect size
[66] López et al, 2014	Case report
[33] Maciel et al, 2012	Microscopic evaluation; lack of data needed to calculate the effect size
[67] Molina-Minao et al, 2009	Sutured wounds, primary intention healing
[68] Ostvar et al, 2015	Lack of data needed to calculate the effect size
[69] Pietramaggiori et al, 2006	Histological and immunohistochemical study
[70] Pietramaggiori et al, 2008	Lack of data needed to calculate the effect size
[71] Sell et al, 2012	In-vitro study
[72] Shayesteh et al, 2012	Experimental lesions on palatal mucosa
[35] Tambella et al, 2014	Spontaneous lesions, not experimentally induced
[73] Tsuzuki et al, 2012	Experimental lesions on epithelium soleare
[74] Vijayaraghavan et al, 2014	Retrospective study
[75] Zubin et al, 2015	Case report

### Risk of bias in included studies

**Fig 2:**
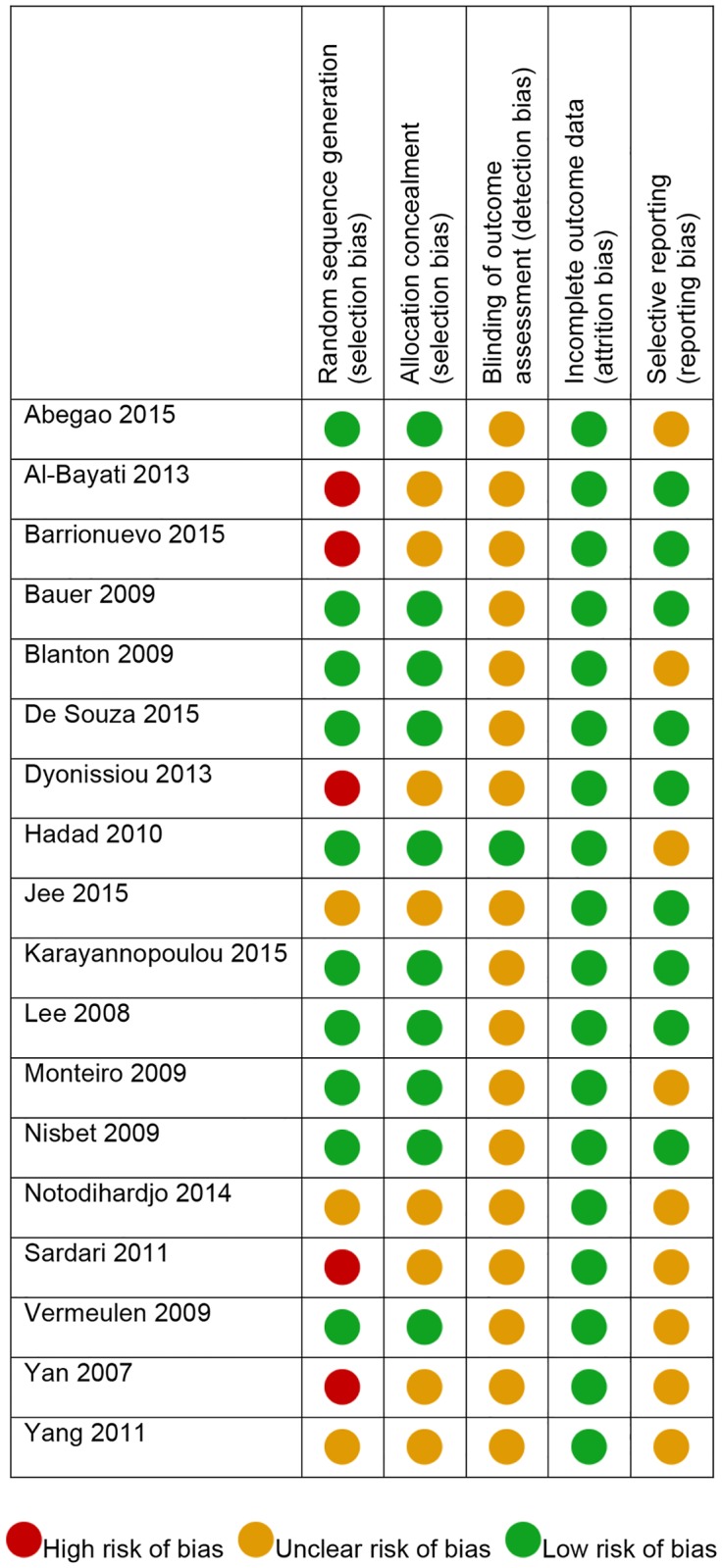
Risk of bias summary. Review author’s judgement obtained by each included study for each type of bias.

Five included studies presented a high risk of selection bias: the result was based on the fact that the allocation of the group was not carried out in a randomised fashion. Only one study reported information on blinding of the evaluators. (Fig 2)

No independent subgroups or multiple comparisons were identified in the included studies, while multiple outcomes and multiple time points (wave) were identified.

For multiple outcomes a conservative approach was adopted, considering the individual outcomes, the results of which are described in the Effects of intervention, meta-analyses 1–3, Figs 3 to 9. The subsequent outcome aggregation is described in the Effects of interventions, meta-analysis 4, Figs 10 to 12.

**Fig 3:**
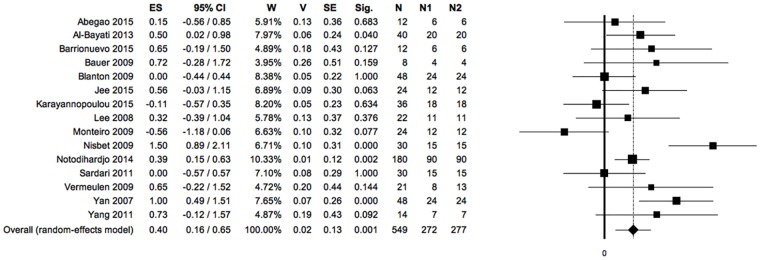
Forest plot: PRP *vs* control, meta-analysis 1: Reduction of open wound area (negative ES, positioned on the left of the null value: favours control; positive ES, positioned on the right of the null value: favours PRP). Heterogeneity analysis: Q = 39.35; df = 14; P = 0.000; I^2^ = 64.42; T^2^ = 0.14; T = 0.37. (ES: effect size; 95%CI: confidence interval; W: weight; V: variance; SE: standard error; Sig: statistical significance (p-value); N: total sample size; N1: sample size PRP group; N2: sample size control group; Q, I_2_, T_2_ and T: indexes of heterogeneity; df: degrees of freedom).

**Fig 4:**
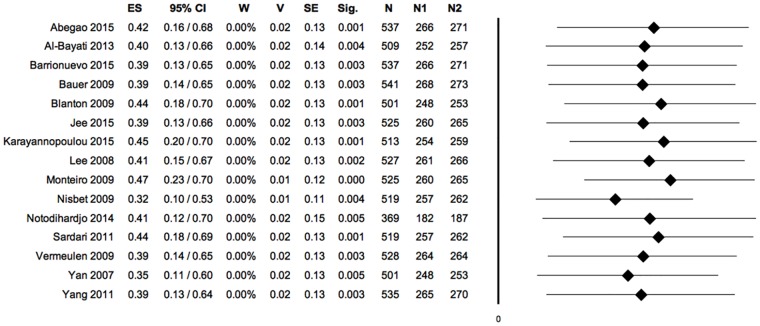
Sensitivity analysis, meta-analysis 1: Reduction of open wound area. (ES: effect size; 95%CI: confidence interval; W: weight; V: variance; SE: standard error; Sig: statistical significance (p-value); N: total sample size; N1: sample size PRP group; N2: sample size control group).

**Fig 5:**
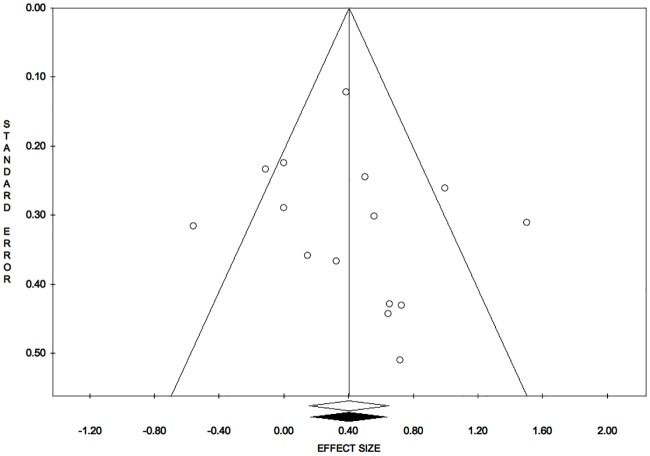
Publication bias analysis, funnel plot, meta-analysis 1: Reduction of open wound area. Trim and fill analysis: trimmed studies = 0. Overall effect size (observed): ES = 0.40; LL = 0.16; UL = 0.65; P = 0.001; V = 0.02; SE = 0.13. Overall effect size (estimated): ES = 0.40; LL = 0.16; UL = 0.65; P = 0.001; V = 0.02; SE = 0.13. Egger’s linear regression test: intercept = 0.60; t = 0.52; P = 0.611.

**Fig 6:**
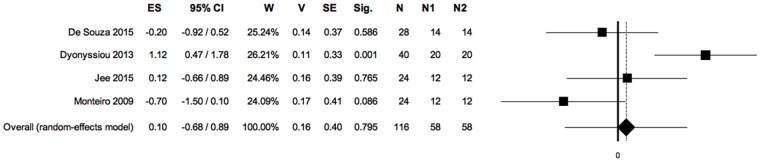
Forest plot: PRP *vs* control, meta-analysis 2: Healing time (negative ES, positioned on the left of the null value: favours control; positive ES, positioned on the right of the null value: favours PRP). Heterogeneity analysis: Q = 13.69; df = 3; P = 0.003; I^2^ = 78.09; T^2^ = 0.50; T = 0.71. (ES: effect size; 95%CI: confidence interval; W: weight; V: variance; SE: standard error; Sig: statistical significance (p-value); N: total sample size; Q, I_2_, T_2_ and T: indexes of heterogeneity; df: degrees of freedom).

**Fig 7:**
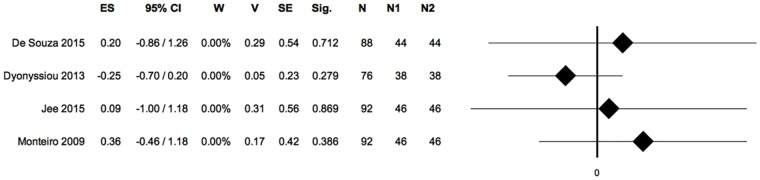
Sensitivity analysis, meta-analysis 2: Healing time. (ES: effect size; 95%CI: confidence interval; W: weight; V: variance; SE: standard error; Sig: statistical significance (p-value); N: total sample size; N1: sample size PRP group; N2: sample size control group).

**Fig 8:**
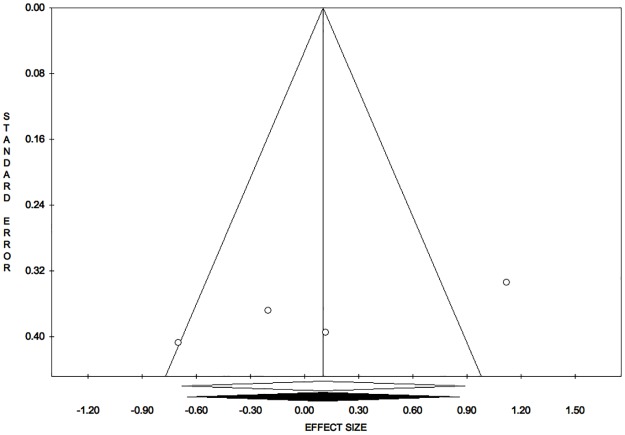
Publication bias analysis, funnel plot, meta-analysis 2: Healing time. Trim and fill analysis: trimmed studies = 0. Overall effect size (observed): ES = 0.10; LL = -0.68; UL = 0.89; P = 0.795; V = 0.16; SE = 0.40. Overall effect size (estimated): ES = 0.10; LL = -0.68; UL = 0.89; P = 0.795; V = 0.16; SE = 0.40. Egger’s linear regression test: intercept = -21.36; t = -2.66; P = 0.117.

**Fig 9:**
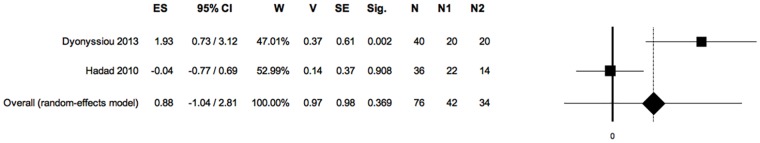
Forest plot: PRP *vs* control, meta-analysis 3: Number of healings (negative ES, positioned on the left of the null value: favours control; positive ES, positioned on the right of the null value: favours PRP). Heterogeneity analysis: Q = 7.59; df = 1; P = 0.006; I^2^ = 86.82; T^2^ = 1.68; T = 1.30. (ES: effect size; 95%CI: confidence interval; W: weight; V: variance; SE: standard error; Sig: statistical significance (p-value); N: total sample size; N1: sample size PRP group; N2: sample size control group; Q, I_2_, T_2_ and T: indexes of heterogeneity; df: degrees of freedom).

**Fig 10:**
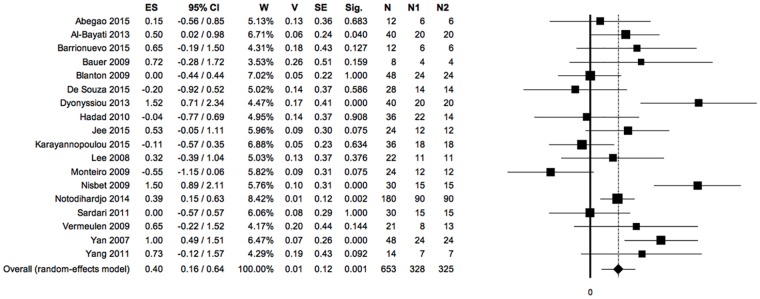
Forest plot: PRP vs control, meta-analysis 4: Combination of all outcomes (negative ES, positioned on the left of the null value: favours control; positive ES, positioned on the right of the null value: favours PRP). Heterogeneity analysis: Q = 50.87; df = 17; P = 0.000; I^2^ = 66.58; T^2^ = 0.16; T = 0.40. (ES: effect size; 95%CI: confidence interval; W: weight; V: variance; SE: standard error; Sig: statistical significance (p-value); N: total sample size; Q, I_2_, T_2_ and T: indexes of heterogeneity; df: degrees of freedom).

**Fig 11:**
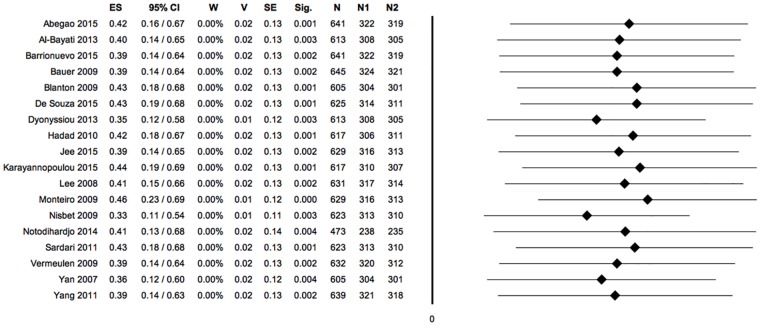
Sensitivity analysis, meta-analysis 4: Combination of all outcomes. (ES: effect size; 95%CI: confidence interval; W: weight; V: variance; SE: standard error; Sig: statistical significance (p-value); N: total sample size; N1: sample size PRP group; N2: sample size control group).

**Fig 12:**
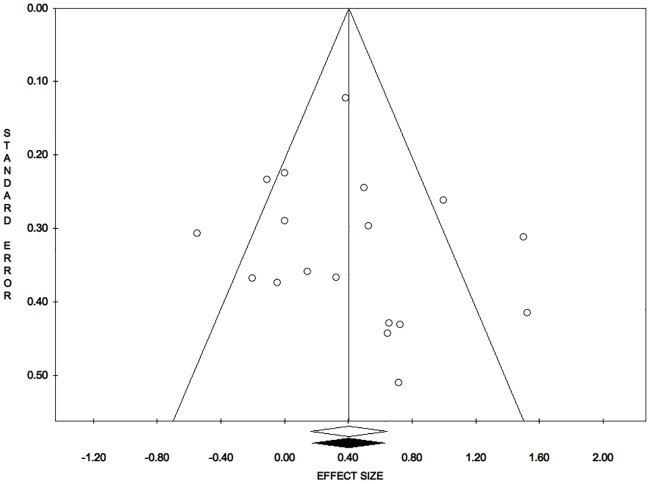
Publication bias analysis, funnel plot, meta-analysis 4: Combination of all outcomes. Trim and fill analysis: trimmed studies = 0. Overall effect size (observed): ES = 0.40; LL = 0.16; UL = 0.64; P = 0.001; V = 0.01; SE = 0.12. Overall effect size (estimated): ES = 0.40; LL = 0.16; UL = 0.64; P = 0.001; V = 0.01; SE = 0.12. Egger’s linear regression test: intercept = 0.63; t = 0.57; P = 0.577.

Before deciding the method to analyse the multiple time points, a pre-analysis was performed to compare the results of the individual waves. The pre-analysis showed that the results of the various waves were similar to each other (ANOVA Q random effect model tests, Q = 3.39, df = 8, P = 0.908), therefore the data of the different waves were combined in meta-analyses.

### Effect of interventions

#### 1) Reduction of open wound area in PRP treated wounds compared to controls (primary outcome)

Fifteen primary studies, considering a total of 549 wounds, reported data on this outcome.

Assessing the reduction in size of open wound area, there was a statistically significant advantage of the PRP-treated group against the control group.

The significance of Q index indicates the presence of heterogeneity among the included studies; the I^2^ index indicates a moderate grade of heterogeneity. (Fig 3)

Although the forest plot in Fig 3 showed two studies with not significant negative ES [51,53], two studies with null ES [30,46], seven studies with not significant positive ES [42,44,45,50,52,56,58] and four studies with significant positive ES [43,54,55,57]: the exclusion of each of the studies, in the sensitivity analysis, would have no relevant effect on the overall results of the meta-analysis supporting the efficacy of PRP for primary outcome. (Fig 4)

The symmetry of the funnel plot, demonstrated statistically by the non-significance of Egger’s linear regression method, associated with the absence of trimmed studies and the overlap between the observed overall ES with the estimated overall ES, showed that the results are not affected by risk of publication bias. (Fig 5)

#### 2) Healing time in PRP treated wounds compared to controls

Four primary studies, considering a total of 116 wounds, reported data on this outcome.

Considering the healing time, there was no statistical difference between the study groups. Two studies showed not statistical negative ES [47,53]; two studies showed positive ES [48,50] among which one with significative statistical difference [48].

The significance of Q index indicates that heterogeneity is present in the primary studies; the I^2^ index indicates a high level of heterogeneity. (Fig 6)

Sensitivity analysis did not influence the overall result of the meta-analysis 2, persisting a lack of statistical difference between study groups. (Fig 7)

The symmetry of the funnel plot, demonstrated statistically by the non-significance of Egger’s linear regression method, associated with the absence of trimmed studies and the overlap between the observed overall ES with the estimated overall ES, showed that the results are not affected by risk of publication bias. (Fig 8)

#### 3) Number of healings in PRP treated wounds compared to controls (number of wounds showing complete healing)

Two primary studies, considering a total of 76 wounds, reported data on this outcome.

Considering the number of wound healings on the total sample size (proportion of healing) a situation of high heterogeneity was found, as evidenced by the Q and I^2^ indices.

The study Dionyssiou 2013 [48] was statistically in favour of treatment with PRP, while the study Hadad 2010 [49] showed a slight favour, although not statistically significant, for the control group. (Fig 9)

As only two studies considered this outcome (meta-analysis 3: Number of healings), the sensitivity analysis (without providing any additional information) proved to be in full agreement with the forest plot.

The publication bias analysis was not possible for this outcome due to the low number of studies.

#### 4) Combination of all outcomes

Considering the aggregation of all outcomes of this meta-analysis (primary and secondary) a statistically significant difference between the two experimental groups appeared (95% CI 0.16,0.64; P = 0.001). PRP treatment generally proved to be more effective in stimulating the healing process of experimental wounds in animals. The overall effect size (= 0.40), interpreted with Cohen’s cut-off values [39], indicated a medium effect size.

The significance of the Q index indicated the presence of heterogeneity among the included studies; the I^2^ index indicated that heterogeneity was moderate. (Fig 10)

The sensitivity analysis showed that the hypothetical exclusion of each one of the experimental studies would not alter the overall results of the meta-analysis performed by combining all the outcomes, in agreement with the hypothesis of favour for the PRP. (Fig 11)

The symmetry of the funnel plot, demonstrated statistically by the non-significance of Egger’s linear regression method, associated with the absence of trimmed studies and the overlap between the observed overall ES and the estimated overall ES, showed that the results are not affected by risk of publication bias. (Fig 12)

### Effect of moderators

The moderator with the greatest influence on the observed heterogeneity was the platelet concentration in the PRP. The platelet concentration recodified from numeric moderator (mean number of platelets/microL) to categorical moderator (mean number of platelets < or ≥ to 1x10^6^/microL) showed a significant difference between the two categories in influencing the effect size, since a concentration of platelets in the PRP ≥ 1x10^6^/microL had a significantly higher effect size (Q = 3.49, P = 0.026). (Table 3)

**Table 3:** Assessment of the moderators’ effect on the combined overall outcome.

	*k*	N_1_	N_2_	Hedge’s *g* [95% CI] Sig.	Q Sig.	I^2^	Contrast Q Sig.
**Country recodified**							1.48
							0.688
Asia	6	159	159	0.57	14.48	65.46	
				[0.22,0.92]	0.013		
				0.001			
Europe	4	58	63	0.33	18.57	83.85	
				[-0.49,1.15]	0.000		
				0.43			
North America	4	81	73	0.33	9.69	69.05	
				[-0.19,0.86]	0.021		
				0.211			
South America	4	30	30	0.25	3.31	9.45	
				[-0.17,0.67]	0.346		
				0.238			
**Animal species recodified**							5.01
							0.125
Non rodents/lagomorphs	10	169	166	0.18	23.26	61.90	
				[-0.11,0.47]	0.005		
				0.233			
Rodents/lagomorphs	8	159	159	0.72	18.62	62.40	
				[0.35,1.10]	0.009		
				0.000			
**Initial wound size recodified**							0.01
							0.907
< 1 cm^2^	5	118	118	0.41	1.42	0.000	
				[0.21,0.61]	0.840		
				0.000			
≥ 1 cm^2^	13	210	207	0.39	49.12	75.57	
				[0.05,0.73]	0.000		
				0.026			
**Funding Source**							1.10
							0.577
No funding	2	12	12	0.36	0.82	0.000	
				[-0.18,0.90]	0.364		
				0.196			
Non-Profit	6	149	149	0.12	11.43	56.24	
				[-0.23,0.47]	0.044		
				0.514			
Profit	5	108	100	0.44	21.55	81.44	
				[-0.15,1.02]	0.000		
				0.142			
**N. of spin**							1.45
							0.228
Double spin	10	205	210	0.53	22.95	60.78	
				[0.24,0.83]	0.006		
				0.000			
Single spin	8	123	115	0.24	21.18	66.95	
				[-0,15,0.62]	0.004		
				0.232			
**Activation procedures**							0.00
							0.944
No activation	6	77	77	0.39	15.41	67.55	
				[-0.09,0.87]	0.009		
				0.108			
Activation	12	251	248	0.41	35.05	68.62	
				[0.12,0.70]	0.000		
				0.006			
**Platelet concentr. recodified**							3.49
							0.026
<1x10^6^/microL	6	99	91	0.14	5.19	3.61	
				[-0.11,0.39]	0.393		
				0.266			
≥1x10^6^/microL	5	155	155	0.70	24.15	83.43	
				[0.17,1.24]	0.000		
				0.010			
**N. of treatment**							0.06
							0.800
Double treatment	5	60	60	0.33	23.34	83.57	
				[-0.35,1.00]	0.000		
				0.342			
Single treatment	13	268	265	0.42	26.40	54.55	
				[0.18,0.66]	0.009		
				0.001			

k: number of studies; N_1_: sample size of PRP group; N_2_: sample size of control group; Hedge’s *g*: effect size, ES; 95% CI: confidence interval 95%; Sig.: statistical significance in double tail; Q: index of heterogeneity Q; I^2^: index of heterogeneity I^2^; Contrast Q: ANOVA Q-test random-effects.

No significant difference in the effect was found for country recodified (Q = 1.48; P = 0.688), animal species recodified (Q = 5.01; P = 0.125), initial wound size recodified (Q = 0.01, P = 0.907), funding source (Q = 1.10; P = 0.577), number of spinning cycles (Q = 1.45; P = 0.228), activation procedures (Q = 0.00; P = 0.944), number of treatments (Q = 0.06; P = 0.800). (Table 3)

### Complications and adverse events during wound healing process

In the control group of a primary study performed on rabbits [48], a clear clinical deterioration was found in 6 of 20 wounds, undergoing a deepening of the wound floor gradually developing into full thickness perforations of the ear pinna; in the PRP group, only one case out of 20 showed delayed healing, in the absence of other complications.

In a primary study performed on horses [53], in the PRP group (7 wounds of 12) a greater tendency to develop exuberant granulation tissue was shown in comparison to the control group (2 of 12 wounds). In addition, the wounds of the PRP group showing this complication needed an average of 3.0 ± 1.37 excisions of exuberant granulation tissue, while in the control group an average of 0.5 ± 0.84 excisions was practiced. However, this difference was not statistically significant (P = 0.19).

No study reported on assessment of pain resulting from injury, life quality, adverse events related to the intervention.

## Discussion

### Summary of main results and quality of the evidence

Based on the growing interest of the scientific community in regenerative medicine, the last few decades have witnessed a significant increase in the number of studies performed both *in vitro* and *in vivo*. These studies have been conducted to develop and validate therapeutic aids, such as PRP, which can potentially influence the natural reparative capacity of tissue. The present systematic review continues this trend, with its purpose to determine if topical application of PRP is able to promote the healing process of experimentally-induced wounds in animals.

The extensive literature search, performed using a variety of different bibliographic databases, shows that the studies included in this review are all very recent, having been published from 2007 to 2016 (28% of them in 2015–2016).

The studies referenced were conducted all over the world, including emerging countries from a scientific point of view, such as Asian countries, which by the way are those that achieved the best results with PRP treatment. Brazil presents the same number of studies (four) as the United States of America (USA). Finally, the European countries, all together, present the same frequency as Brazil and USA.

Regarding the geographical distribution of the financed studies, the studies reporting sources of funding were performed in the USA (3 for profit; 1 non-profit), Greece (2 for profit), France (1 non-profit), Brazil (1 non-profit) and Asia (Japan, Iran, Korea, each with 1 non-profit).

During the systematic review, two types of complex meta-analytical database were detected: multiple outcomes and multiple time points.

For multiple outcomes a conservative approach was adopted, in order to respect the complexity of primary studies. Only at a later stage, an aggregation of outcomes was performed to obtain a comprehensive synthesis of the results.

The overall findings of the meta-analysis are suggestive of a positive effect of PRP, but do not support completely the hypothesis of superiority of the group treated with PRP compared to the control group, since primary and combined outcome measures showed statistically significant differences but secondary outcome measures did not.

The primary outcome evaluation (reduction of open wound area in PRP treated wounds compared to controls) indicates a statistically significant difference between the study groups with advantage of the treated group. This finding is associated with a moderate degree of heterogeneity. The quality of evidence obtained in the meta-analysis for the primary outcome was demonstrated by sensitivity analysis, excluding alternately each of the studies, even showing negative ES [51,53], null ES [30,46], or statistically positive ES [43,54,55,57]: no relevant changes occurred in the results of the meta-analysis of primary outcome, persisting in statistical agreement with the hypothesis that supports the superiority of the PRP group. The sensitivity analysis finds its own indication in the identification of potential "outlier studies". This term indicates studies whose results are extremely different from those reported in other studies. The result of primary outcome analysis is confirmed by the absence of evidence of risk of publication bias.

The evaluation of outcome 2 (healing time) shows a heterogeneous balancing between studies that favoured PRP group and studies that favoured control group. Only one study reported a significantly lower healing time in PRP group [48]. Both studies showing negative ES in this outcome were performed on horses [47,53]. It is widely described in literature that second intention wound healing in equidae may be more complicated than in other animal species. The development of exuberant granulation tissue is a common cause of delayed healing in equine limb wounds [76,77]. As reported by Monteiro et al., topical application of PRP in small granulating wounds could favor an excessive formation of granulation tissue and delay the healing of limb wounds in horses [53]. This aspect should be considered when planning clinical trials or treatments in equine wounds. The results for outcome 2 are not affected by risk of publication bias. Despite the high degree of heterogeneity, there were no outlier studies for this outcome.

Considering the number of wounds completely healed in PRP group compared to control group (number of healings, outcome 3), a condition of high heterogeneity is detected, as indicated by the Q and I^2^ indices, and no significant difference between the two groups is observed. The Dionyssiou et al. (2013) study [48] is significantly in favour of the treatment with PRP; on the contrary, the study by Hadad et al. (2010) [49] is not-significantly in favor of the control group. The analysis of publication bias was not possible for this outcome, since only two primary studies are reported.

The general meta-analysis, obtained considering all the outcomes (primary and secondary), shows a statistically significant difference between the two experimental groups with a moderate degree of heterogeneity. The comprehensive meta-analysis agrees with the hypothesis that supports a greater efficacy of the PRP treatment on the healing process of experimentally induced wounds in animals. The sensitivity analysis confirms the quality of the evidence; in fact, the exclusion of each of the primary experimental studies does not produce any changes in the final results of the meta-analysis. The general absence of publication bias provides further support to the quality of the evidence.

Both the secondary outcomes (healing time and number of healings) had a smaller impact on the result of the combination of all outcomes than the primary outcome (reduction of wound area). The much higher number of wounds and primary studies considered, as well as the higher number of calculated ES, explain the greater impact of the primary outcome on the combined outcome and on the overall results of the meta-analysis.

The principal strengths of the study are the fairly high number of studies included in the analysis and the general absence of publication bias. The main limitation relates to the modest average number of wounds in the primary studies, the moderate/high degree of heterogeneity, the variety of animal species considered, the type of control treatment and the fact that PRP is a biologic product that, for its own nature, could lead to many uncontrollable variables.

In this systematic review, different animal species have been considered with the aim of obtaining a global assessment of the effect of PRP on animals, but it could also be a limiting factor since the different species may have different healing pattern.

In all included studies, control wounds underwent placebo application (saline solution, milli-Q-water) or were left untreated. This aspect was not analyzed among moderators because of the substantial homogeneity in the therapy of control group. However, it is necessary to recognize that comparing a therapy with sub-standard control treatment, such as placebo or non-treatment, can lead to an overestimation of the effect of treatment under investigation. Future prospective studies should compare PRP to therapies or advanced dressings that truly support wound healing.

Several moderators were taken into consideration to explain the heterogeneity observed in the studies included in the quantitative synthesis. These moderators included the country where the study was performed (categorical moderator recoded at 4 levels by geographic area corresponding to the continents). The animal species (dichotomous categorical moderator, including rodent/lagomorph mammals and non-rodent/lagomorph mammals) was considered one of the main moderators because of the substantial differences amongst the animal species considered. The initial wound size recodified (continuous moderator variable recoded and expressed as a dichotomous variable, wound size < or ≥ 1 cm^2^) was considered because it could potentially exert a great influence on the wound-healing process, though this could not be confirmed in this meta-analysis. Current literature considers the source of funding as one of many possible causes of bias in scientific research and associated with differences in research report quality [78–80]. On that basis, the source of funding was analysed as moderator (categorical moderator with three levels: for profit, for non-profit, and no-funding statement) but it did not show any significant effect in this meta-analysis. Studies not reporting any source of funding (n = 5) were considered as missing-data and were therefore excluded from the analysis. Some important technical parameters of production process and therapeutic application were considered as moderators, such as the number of spinning cycles used to obtain the PRP (categorical moderator with two levels, single or double centrifugation); the application of PRP activation procedures (categorical moderator with two levels, use or non-use of PRP activation procedures); the PRP platelet concentration (continuous moderator variable recoded and expressed as a dichotomous variable, concentration of platelets < or ≥ 1x10^6^ / microL); the number of treatments (categorical moderator with two levels, single or multiple treatment). The most influential moderator was the PRP platelet concentration. Theoretical and mathematical grounds justified the decision to recode this moderator. The theoretical ground was motivated by the finding that some authors identified the concentration of 1x10^6^ platelets/microL as a "reasonable compromise" of reference to determine the quality of the product to obtain a therapeutic effect [81]. The mathematical ground was motivated to achieve a uniform distribution rate of the studies in each of the recoded moderator levels. The analysis showed a significant difference between the two categories in influencing the effect size; in fact, the platelet concentration greater or equal to the recoded cut-off showed an effect size significantly higher compared to the lower platelet concentration. Although a close linear correlation has not been established [81,82], this finding seems to support the hypothesis of some authors who relate the platelet concentration to the clinical effect as it is positively correlated with the concentration of growth factors [14,15,83]. Moreover, an excessively high platelet concentration could be counterproductive to the healing process, as potentially able to inhibit the angiogenic process [84]. The choice to use as moderator the platelet concentration in PRP (reported by 11 studies [30,43,45–51,54,55]), instead of platelet fold increase from whole blood to PRP (reported by 5 studies [44,45,51,53,54]), is supported by scientific literature [81]. Platelet concentration of normal whole blood could differ substantially between animal species, therefore it seems reasonable to think that, considering only the fold increase in platelets, some difficulties in standardisation of PRP and in interpretation of results may arise. The platelet fold increase could be considered a first rough measure of the quality of the production process; conversely, the platelet concentration could be considered a first rough measure of the quality of the product (PRP) for clinical use. To fully interpret this aspect, any researcher carrying out a study on PRP should report both of these data.

Five studies had a high risk of selection bias [30,43,44,48,57]; this result, however, must be interpreted on the basis of the type of studies. Based on the inclusion criteria, experimental clinical studies characterised by skin lesions induced in animals are considered in this review. Each study presented an artificial-iatrogenic, experimental condition of homogeneity between treated and control groups. In the 5 studies with a high risk of selection bias, this data was correlated to the lack of randomisation in the sequence generation, as the researchers arbitrarily assigned experimental groups (i.e. left side wound always assigned to a control group, or *vice versa*). This randomisation issue could be considered less influential in this type of study, because they are performed on experimentally-induced injuries. On the other hand, clinical trials are performed on patients with spontaneous lesions and usually start from a natural condition of inhomogeneity in terms of quality and quantity of injuries; therefore, they require a low selection bias to increase the clinical evidence and to be legitimately contemplated in a meta-analysis.

### Overall completeness and applicability of evidence

The use of PRP is undergoing a considerable increase in recent years, as evidenced by the number of studies considered in this and other systematic reviews [85–90].

In this review, experimental skin lesions induced in animals were examined. This condition undoubtedly presents a high level of experimental evidence, both at the beginning of the study (since these lesions are substantially identical from a morphological and qualitative point of view) and during the study, maintaining the same conditions of homogeneity between groups during the follow-up. However, they are experimentally induced in healthy patients. Therefore, they have to be considered acute lesions on an ideal substrate with normal healing potential conditions in which the advantage deriving from PRP might be rather limited in clinical practice.

According to literature, clinical use of PRP in wounds could represent a benefit to patients suffering from difficult-to-heal wounds. Although this type of injury was not the subject of this meta-analysis, patients displaying chronic wounds, with poor healing tendencies (for example wounds, sores and pressure ulcers, diabetic and vascular ulcers), which are systematically characterised by a state of chronic inflammation and a GFs deficit [3,4], could benefit even more from treatment with PRP [35,53]. In partial agreement with this hypothesis is a clinical study performed in dogs with spontaneous chronic decubital wounds. The authors found that wounds older than 14 weeks had a greater reduction in wound size after PRP treatment than wounds that were <14 weeks. However, they did not find significant difference in complete wound healing [35]. A systematic review reported a low quality of the evidence to suggest autologous PRP for treating human chronic wounds; current evidence is based on a small number of randomised clinical trials with a high or unclear risk of bias, and concludes that well-designed and adequately powered clinical trials are needed [90].

### Potential biases in the review process

In some cases it was not possible to use the original data. Some authors did not provide the requested data; therefore, only available data have been used for the effect size calculation. The studies with a null effect size (ES = 0.00), to which an input significance P = 1.0 was attributed [30,46], were anyway considered in the quantitative analysis following the more conservative approach to data. The overall results of the meta-analysis were not substantially affected by these studies, as shown by the sensitivity analysis performed on each primary, secondary and aggregate outcome.

## Conclusions

### Implications for practice

The overall findings of this systematic review are suggestive of a positive effect of PRP in the treatment of experimentally-induced skin wounds, but do not support completely the hypothesis of superiority of the group treated with PRP compared to the control group. Wound area reduction and combined outcome measures were positively affected by PRP, while healing time and number of healed wounds were not. PRP can be considered an effective adjunctive therapy in stimulating second intention healing of acute wounds in healthy animals. PRP-products containing concentrations of platelets ≥1x10^6^/microL seem to have better effect than those with lower concentrations.

### Implication for research

Based on the results of this systematic review, well-designed, large-scale RCT on spontaneous wounds are needed to determine whether using PRP represents a benefit in clinical conditions. In such clinical studies, it will be necessary to define some key elements for the interpretation of results, such as the technique used for PRP production and activation, method and time of administration and characteristics of the lesion. Currently in veterinary medicine, there is only one RCT performed using PRP on chronic pressure ulcers in dogs [35] and the results are very encouraging. In addition to the studies on acute and chronic spontaneous wounds, more clinical trials on patients affected by conditions of wound healing difficulties, such as endocrine disorders, degenerative diseases, cytostatic and corticosteroid therapies, may provide additional and valuable evidence on the use of PRP in veterinary medicine.

## Supporting information

S1 Table PRISMA checklist. (PDF)
